# Humoral immune response induced with dengue virus-like particles serotypes 1 and 4 produced in silkworm

**DOI:** 10.1186/s13568-022-01353-6

**Published:** 2022-01-31

**Authors:** Doddy Irawan Setyo Utomo, Sabar Pambudi, Enoch Y. Park

**Affiliations:** 1 Laboratory of Biotechnology, Department of Bioscience, Graduate School of Science and Technology, Shizuoka University, 836 Ohya, Suruga-ku, Shizuoka, 422-8529 Japan; 2 Center of Pharmaceutical and Medical Technology, National Research and Innovation Agency (BRIN), Jl. Kawasan Puspiptek, Gedung I LAPTIAB, Kota Tangerang Selatan, Banten 15314 Indonesia; 3 Laboratory of Biotechnology, Research Institute of Green Science and Technology, Shizuoka University, 836 Ohya, Suruga-ku, Shizuoka, 422-8529 Japan

**Keywords:** Dengue virus, Capsid, Premembrane, Envelope, Dengue virus-like particle, Silkworm

## Abstract

Dengue is an arboviral disease, which threatens almost half the global population, and has emerged as the most significant of current global public health challenges. In this study, we prepared dengue virus-like particles (DENV-LPs) consisting of Capsid-premembrane-envelope (CprME) and premembrane-envelope (prME) polypeptides from serotype 1 and 4, which were expressed in the silkworms using *Bombyx mori* nucleopolyhedrovirus (BmNPV) bacmid. 1CprME, 1prME, 4CprME, and 4prME expressed proteins in hemolymph, and the molecular weight of the purified proteins was 55 kDa, respectively. The purified polypeptides formed spherical Dengue virus-like particles (DENV-LPs) with ~ 30–55 nm in diameter. The immunoelectron microscopy (IEM) images revealed antigens to the surface of a lipid bilayer of DENV-LPs. The heparin-binding assay shows a positive relationship between absorbance and E protein domain III (EDIII) quantity, which is supported by the isothermal titration calorimetry assay. This indicates a moderate binding affinity between heparin and DENV-LP. The high correlation between patient sera and DENV-LP reactivities revealed that these DENV-LPs shared similar epitopes with the natural dengue virus. IgG elicitation studies in mice have demonstrated that DENV-LPs/CPrMEs elicit a stronger immune response than DENV-LP/prMEs, which lends credence to this claim.

**Supplementary Information:**

The online version contains supplementary material available at 10.1186/s13568-022-01353-6.

## Highlights

- Dengue virus-like particles for serotype 1 and serotype 4 (DENV-LPs/1 and DENV-LPs/4) were produced in silkworm.
- Heparin-binding assay by ELISA and ITC showed that DENV-LPs/1 and DENV-LPs/4 contain Envelope Domain III.
- DENV-LPs/1 and DENV-LPs/4 showed affinity to sera from human dengue patients and immunized mice.

**Supplementary Information:**

The online version contains supplementary material available at 10.1186/s13568-022-01353-6.

## Introduction

Dengue fever is a significant public health issue that has been reported in the Americas, Africa, Southeast Asia, Europe, the Western Pacific, and the Eastern Mediterranean. This arboviral disease is endemic in more than 100 countries, and approximately 96 million infected individuals have symptoms of varying severities. There has been a growing public health concern about dengue fever in the last few decades. The World Health Organization (WHO) named it one of the top ten global health threats in 2019, highlighting the critical need for a safe and effective vaccine. Despite numerous attempts, identifying the best candidate for a dengue vaccine continues to be a difficult task due to some critical factors that must be considered (Bhatt et al. 2021; Redoni et al. 2020).

Dengue virus (DENV) is the etiological agent for dengue fever. DENV belongs to the Flaviviridae family and Flavivirus genus. It has four antigenically distinct serotypes and antigenically similar yet genetically diverse.. Adaptive immunity induced by one DENV serotype does not provide long-term protection against infection with the other three heterotypic DENVs There is 70% sequence homology between DENV serotype 1–4 (DENV-1–DENV-4), which means that numerous immunogenic epitopes are conserved at some level. DENV is an enveloped virus with a single positive-strand RNA genome that encodes three structural proteins, Capsid (C), premembrane (prM), and envelope (E), as well as seven nonstructural proteins (NS1, NS2A, NS2B, NS3, NS4A, NS4B, and NS5) (Redoni et al. 2020; Yousaf et al. 2018).

Based on a 70-year study that examined the spread of DENV worldwide, the most reported strains were DENV-1, DENV-2, and DENV-3, and the least frequently recorded was DENV-4. Although DENV-4 was the first serotype of dengue to diverge in phylogenetic investigations of the Flavivirus genus, it spread slowly worldwide. DENV-1 and DENV-4 cause dengue fever with different degrees of severity. When DENV-1 infection was compared to DENV-4 infections, the duration of fever, which essentially correlates with the severity of the illness, was much longer for DENV-1. DENV-1 infection was also associated with more severe clinical manifestations than DENV-4 infection. Primary DENV-4 infection is a relatively mild sickness, but primary DENV-1 infection has more severe symptoms (Nishiura and Halstead 2007; Sang et al. 2019; Villabona-Arenas and Zanotto 2013).

Virus-like particles (VLPs) are viruses with a shell but no genetic material. VLPs may mimic the organization and conformation of wild viruses because they contain multiprotein determinants. The organization and conformation of native viruses can be used to explore virus infection mechanisms and stimulate the host immune system to produce robust immune responses (Roldao et al. 2010). Furthermore, VLPs do not cause infections because they lack the viral genome. These properties of VLPs make them potential vaccine candidates that may be more efficient and safer than conventionally attenuated or inactivated viruses (Noad and Roy 2003).

Two successful approaches have been applied to produce recombinant VLPs of flaviviruses, including capsid-premembrane-envelope (CprME) protein genes and pre-membrane-envelope (prME) proteins in *cis* as well as in *trans* from plasmid vectors. Both types of approaches should result in the formation of particles. Although the C protein is not required to form particles, the inclusion of C protein may have a stabilizing effect on VLP assembly. Capsid proteins can be arranged in one, two, or three layers, depending on their size. Some single-layer VLPs can contain more than one structural protein, whereas others cannot. When compared to the structure of single-protein VLPs (which is relatively simple), multiprotein VLPs (which contain several distinct capsid layers) have additional structural components (Krol et al. 2019; Nooraei et al. 2021).

Baculovirus expression vector system (BEVS) is a high-level mass production tool for recombinant proteins in silkworm. This enables us to express eukaryotic recombinant proteins with posttranslational modifications similar to mammals’. The number of recombinant proteins produced by silkworm-BEVS in silkworm larvae is frequently significantly higher than that produced by Sf9-BEVS in cultured cells (Kato et al. 2010; Vipin Kumar Deo 2012). Feeding silkworms is exceptionally inexpensive, with a total cost of approximately USD 20 for twenty larvae. Thus, it costs slightly more than USD 20 to obtain approximately 1 mg of active Protein kinase B alpha (PKBα) (Maesaki et al. 2014). This approach is comparable in cost to an expression system based on *Escherichia coli*. Furthermore, because silkworms have low-cost productivity equal to the *E. coli* expression system, protein production can be quickly and inexpensively scaled up. This expression system is favorable for vaccine development (Fujita et al. 2020).

Previously, we have successfully expressed DENV-LPs serotype 2 (DENV-LP/2CprME and /2E) and DENV-LPs serotype 3 (DENV-LP/3prME and /3CprME) by removing capsid-anchor in the C region (Utomo et al. 2019, 2020). In this study, we prepared DENV-LPs consisting full-length of Capsid-Premembrane-Envelope (1CprME and 4CprME) and Premembrane-envelope (1prME and 4prME) polypeptides from serotypes 1 and 4, which were expressed using *Bombyx mori* nucleopolyhedrovirus (BmNPV) bacmid in silkworms. We observed the formation of VLPs, confirmed envelope domain III (EDIII) of DENV using a heparin-binding assay by ELISA and ITC, reactivity toward dengue patient sera, and demonstrated the elicitation of antibody production in a mouse model.

## Materials and methods

### Construction and preparation of recombinant BmNPVs

The C-prM-E and prM-E polypeptide coding sequences (GenBank: DENV-1 KM204119, DENV-4 KR011349, Genewiz, New Jersey, USA) were used. A linker sequence (GGGGSGGGGS) and HA-tag sequence (YPYDVPDYA) for DENV-1 constructs, a Strep tag-II sequence (WSHPQFEK) for DENV-4 constructs, and a FLAG-tag sequence (DYKDDDDK) for both serotype constructs were fused at the C-terminus using a template (the synthetic gene described above) by polymerized chain reaction (PCR) with a template for the coding sequence of DENV-1CprME and DENV-4CprME. Used primers were 1CprME-F, 1CprME-R-EcoRI, 4CprME-F, 4CprME-R-EcoRI (Table 1). To isolate the DENV-1prM-E and DENV-4prM-E coding sequences, a primer set (1prME-F, 1prME-R-EcoRI, 4prME-F, 4prME-R-EcoRI, Table 1) was used. PrimeSTAR Max DNA Polymerase (TaKaRa, Kyoto, Japan) has been used for the PCR process with composition 25 μl PrimeSTAR Max Premix (2×), 10 pmol forward primer, 10 pmol reverse primer, 200 ng DNA template, and sterilized ddH_2_O up to 50 μl. The PCR steps began with an initial denaturation at 98 °C for 10 s, followed by 35 cycles of 98 °C for 10 s, 55 °C for 5 s, and 72 °C for 20 s, and finished with 72 °C for 3 min for the final extension. The PCR process was carried out using a thermal cycler (TaKaRa). Each construct was ligated into the pFastbac1 vector (Thermo Fisher Scientific K. K., Tokyo, Japan), and the resulting vector was transformed into *E. coli* BmDH10bac CP^−^ Chi^−^. BmNPV/1CprME, BmNPV/4CprME, BmNPV/1prME, and BmNPV/4prME bacmids were isolated from white colonies. Ten or twenty μg of each recombinant BmNPV bacmid DNA was mixed with 0.1% chitosan (Sigma–Aldrich, Tokyo, Japan) and 2% (w/v) of 2-(*N*-morpholino) ethanesulfonic acid (MES) buffer (Sigma–Aldrich). The recombinant BmNPV bacmid DNA mixture was incubated for approximately 45 min at room temperature (RT) before injection. Subsequently, ~ 50 μl of the mixture was injected into a fifth-stage instar silkworm larva (Ehimesansyu, Ehime, Japan). The hemolymph containing recombinant BmNPV was collected from the larvae 6–7 days post-injection (dpi) and mixed with 1-phenyl-2-thiourea at 1 mM, with aliquots of hemolymph stored at – 80 °C before use (Boonyakida et al. 2021; Suhaimi et al. 2019; Utomo et al. 2019, 2020).

**Table 1:** Used primers

Name	5′–3′
1CprME-F	TAA TGG ATC CAT GAA CAA TCA GCG CAA A
1CprME-R-EcoRI	TAA TGA ATT CTC AAG CGT AGT CCG GAA CA
1prME-F	TAA TGG ATC CAT GTT TCA CCT GAC CAC CAG GGG
1prME-R-EcoRI	TAA TGA ATT CTC AAG CGT AGT CCG GAA CA
4CprME-F	TAA TGG ATC CAT GAA CCA GAG GAA AAA AGT
4CprME-R-EcoRI	TAA TGA ATT CTC ATT TTT CGA ACT GGG GG
4prME-F	TAA TGG ATC CAT GTT CCA TCT CTC GAC GCG TGA T
4prME-R-EcoRI	TAA TGA ATT CTC ATT TTT CGA ACT GGG GG
pFastBac1-F	TAT TCC GGA TTA TTC ATA CC
pFastBac1-R	ACA AAT GTG GTA TGG CTG ATT
pUC/M13-F	CCC AGT CAC GAC GTT GTA AAA CG
pUC/M13-R	AGC GGA TAA CAA TTT CAC ACA GG

Underlines indicate restriction enzyme cleavage sites

### Expression and purification

Ten fifth-stage instar silkworm larvae (Ehimesansyu) were injected with 100-fold diluted hemolymph in phosphate-buffered saline (PBS) (137 mM NaCl, 2.7 mM KCl, 8 mM Na_2_HPO_4_, and 2 mM KH_2_PO_4_, pH 7.4). They were fed a synthetic diet (Silkmate S2, Nosan Co., Yokohama, Japan). After 5 dpi, the hemolymph was collected by cutting the larva’s leg. The hemolymph was diluted tenfold with 50 mM Tris–HCl (pH 7.5) containing 300 mM NaCl before being applied to DDDDK-tagged protein purification gel column chromatography (Medical & Biological Laboratories, Nagoya, Japan). The column was washed with 50 mM Tris–HCl (pH 7.5) containing 300 mM NaCl. The proteins were eluted with 0.17 M glycine–HCl buffer (pH 2.3). The eluents were neutralized immediately with 1 M Tris–HCl (pH 8.0). Ultrafiltration was used to concentrate the eluents using an Amicon Ultra-0.5 mL Centrifugal Filter Unit with an Ultracel-30 K membrane (Merck Japan, Tokyo, Japan). A BCA protein assay kit was used to determine the protein concentration (Thermo Fisher Scientific K. K.).

### Western blot analysis

To separate the proteins, 10% or 12% sodium dodecyl sulfate–polyacrylamide gel electrophoresis (SDS-PAGE) was used and subsequently subjected to western blotting by blotting the proteins trapped in acrylamide gel onto an Immobilon-P PVDF (polyvinylidene fluoride) membrane (Merck Japan) using the Mini Trans-Blot Electrophoretic Transfer Cell (Bio-Rad, Hercules, CA, USA). After blotting, the membrane was blocked in Tris-buffered saline with 0.1% Tween 20 detergent (TBST) (pH 7.6) with 5% skim milk (FUJIFILM Wako Pure Chemical). Then the membrane was incubated in 10,000-fold diluted mouse anti-HA tag antibody (Medical and Biological Laboratories) for DENV-1 constructs and anti-strap tag II antibody (Medical and Biological Laboratories) for DENV-4 constructs. Alternatively, 1500-fold diluted specific serotype monoclonal anti-envelope antibodies, anti-E DENV-1 E29 clone (BEI Resources, Virginia, US) for DENV-1 constructs, and anti-E DENV-4 E42 clone (BEI Resources) for DENV-4 constructs were used as the primary antibodies. Anti-E DENV-2 3H5-1 clone (BEI Resources) and anti-E DENV-3 E1 clone (BEI Resources) antibodies were also used to assess cross-reactivity between serotypes for all purified proteins. C protein was also confirmed using an anti-capsid polyclonal antibody (GeneTex, CA, USA). After three washes with TBST, the membrane was incubated for one hour with 10,000-fold diluted anti-mouse IgG antibody conjugated to horseradish peroxidase (HRP) (FUJIFILM Wako Pure Chemical). The specific bands were discovered using a Fluor-S MAX Multi-Imager (Bio-Rad).

### Heparin-binding assay by ELISA

The heparin-binding assay by ELISA was performed with modifications as previously described (Utomo et al. 2019, 2020). Six ng/ml diluted biotin-labeled heparin (Sigma-Aldrich Japan) and 1.8 ng of heparin were immobilized into blockless avidin plate (Sumitomo Bakelite, Tokyo, Japan) wells and washed three times with PBS. For negative control, 2 μg of BSA was used. Purified proteins of 1CprME, 1prME, 4CprME, and 4prME at various concentrations (0.5, 1, 5, and 10 g/ml) were added to wells at corresponding quantities, incubated at room temperature for one hour, and then washed with PBST. After serial washing, a 1000-fold diluted rabbit anti-DENV E polyclonal antibody (GeneTex) was added, followed by a 1000-fold diluted HRP-conjugated anti-rabbit IgG antibody (FUJIFILM Wako Pure Chemical). To stop the reaction, 100 μl of substrate 0.1 mg/ml 3.3′,5.5′-tetramethylbenzidine (TMB) in 100 mM sodium acetate (CH_3_COONa), pH 6.0, was added to each well with 0.2% (v/v) 30% hydrogen peroxide, and 50 μl of 1 N H_2_SO_4_ was added. The absorbance was estimated at 450 nm.

### Isothermal titration calorimetry assay for heparin-binding to DENV-LPs

The binding affinities of DENV-LP to heparin were determined using isothermal titration calorimetry (ITC) (MicroCal iTC200, Malvern Panalytical Ltd, Enigma Business Park, UK). Titrations were performed at a temperature of 25 °C by injecting 2 μl aliquots of 1000 μM ligand dissolved in 1 × PBS buffer into a cell containing 10 μM DENV-LP. The heat release was recorded, and the titration data were analyzed with MicroCal Origin ITC software (Malvern Panalytical Ltd). Thermodynamic parameters were determined by fitting experimental data with nonlinear least-squares using the one-set sites binding model (Duff et al. 2011; Ikegaya et al. 2021).

### Human DENV-infected sera interaction with DENV-LPs

Direct ELISA was carried out with modifications as previously described (Utomo et al. 2019, 2020). An interaction between antigens, 1CprME, 1prME, 4CprME, and 4prME, and patient sera were detected using a direct ELISA method. Dengue patient sera [rapid diagnostic test NS1(+)] were used. Sera were obtained from the Centre of Pharmaceutical and Medical Technology, National Research and Innovation Agency, Jakarta, Indonesia. Protocols of the collection were reviewed and approved by the Health Research Ethics Committee-University of Indonesia and Cipto Mangunkusumo Hospital (HREC-FMUI/CMH) (approval no. KET-1358/UN2.F1/ETIK/PPM.00.02/2020).

Each diluted sample of 1CprME, 1prME, 4CprME, and 4prME, 100 µl of 20 ng/ml in coating buffer (0.05 M carbonate-bicarbonate, pH 9.6), was applied to a 96-well ELISA plate and incubated overnight at 4 °C. After incubation, the coating solution was discarded, and a 100 µl blocking solution (5% skim milk in PBS) was added to each well, followed by 1 h at 37 °C incubation. The plates were then washed serially with PBST buffer before adding 100 µl of 1:50 patient sera in PBS. Plates were then incubated at 37 °C for 1 h before being washed three times with washing buffer and before the addition of 100 µl of 1:5000 anti-human IgG-HRP conjugated antibody. Plates were then incubated at 37 °C for 1 h and washed, and 50 µl of TMB substrate was applied and incubated for 10 min before being stopped with 50 µl of 1 M H_2_SO_4_. The absorbance was measured at 450 nm.

### Immunoelectron microscopy

Immunoelectron microscopy (IEM) was carried out as previously described (Utomo et al. 2020) with modifications. The purified antigen sample was added to the Cu-grid transmission electron microscopy (TEM) (Nisshin EM Co., Ltd., Tokyo) and incubated for 30 s at room temperature, washed with 30 µl of PBS, and incubated for 30 s, repeated three times. BSA (30 µl of 2% v/v) was used for blocking after adding a purified antigen sample and washed three times with PBS. The Cu-grid was washed in stages. The first and secondary antibodies were anti-E rabbit polyclonal antibody (FUJIFILM Wako Pure Chemical) diluted 30 times, and goat anti-rabbit IgG-conjugated (FUJIFILM Wako Pure Chemical) gold nanoparticles diluted 50 times, respectively. The Cu grid was treated with 1% phosphotungstic acid and analyzed with a JEM-2100F TEM system (JEOL Ltd., Tokyo, Japan).

### Mice immunization

Mouse immunization was carried out as previously described (Utomo et al. 2020) with modifications. In this study, 50 BALB/c mice (PT Indoanilab, Bogor, Indonesia) aged 4–6 weeks were divided into ten groups: (i) negative control (PBS), (ii) immunized with alhydrogel as an adjuvant, (iii) immunized with 1CprME, (iv) immunized with 1CprME + adjuvant, (v) immunized with 1prME, (vi) immunized with 1prME + adjuvant, (vii) immunized with 4CprME, (viii) immunized with 4CprME + adjuvant, (ix) immunized with 4prME, and (x) immunized with 4prME + adjuvant. All mice were kept in a temperature-controlled, light-cycled room and were divided into ten groups based on the immunogen. Each mouse was immunized three times intraperitoneally within 2 weeks with 50 μg of purified 1CprME, 1prME, 4CprME, and 4prME proteins with alhydrogel adjuvant. Blood samples were collected via the tail vein on Days 0, 16, and 30, and sera were isolated and stored at – 80 °C. All animal procedures were conducted in compliance with established guidelines from the Animal Laboratory of Center of Pharmaceutical and Medical Technology, National Research and Innovation Agency, Indonesia. Animal experiment protocols were reviewed and approved by the Health Research Ethics Committee-University of Indonesia and Cipto Mangunkusumo Hospital (HREC-FMUI/CMH) (approval no. KET-721/UN2.F1/ETIK/PPM.00.02/2021). The interaction between antigens, 1CprME, 1prME, 4CprME, and 4prME, with the respective mice sera, was detected using a direct ELISA method.

One hundred µl of 20 ng/ml diluted antigen, respectively, in coating buffer (0.05 M carbonate-bicarbonate, pH 9.6), was applied to a 96-well ELISA plate and incubated overnight at 4 °C. After incubation, the coating solution was discarded, and a 100 µl blocking solution (5% skim milk in PBS) was added to each well, followed by 1 h at 37 °C incubation. The plates were then washed serially with PBST buffer before adding 100 µl of 1:10 mice sera in PBS. Plates were then incubated at 37 °C for 1 h before being washed three times with washing buffer and before the addition of 100 µl of 1:5000 anti-mouse IgG-HRP conjugated antibody. Plates were then incubated at 37 °C for 1 h and washed, and 50 µl of TMB substrate was applied and incubated for 10 min before being stopped with 50 µl of 1 M H_2_SO_4_. The absorbance was measured at 450 nm.

## Results

### Expression of 1CprME, 1prME, 4CprME and 4prME polypeptides in silkworm

**Fig. 1:**
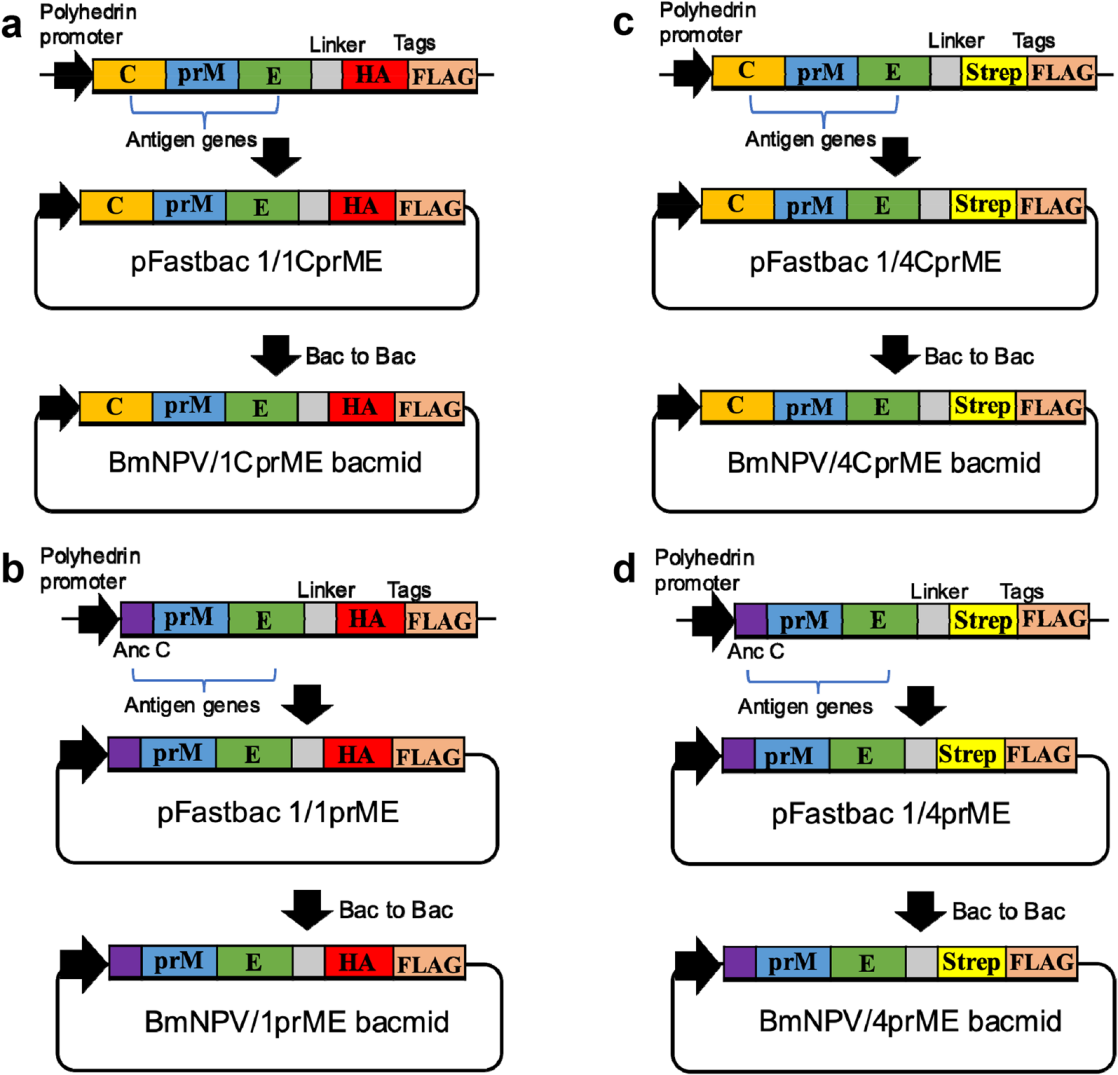
Construction of recombinant dengue virus structural proteins expressed in this study. **a** 1CprME, **b** 1prME, **c** 4CprME, and **d** 4prME polypeptides of DENV-1 and 4 were expressed in silkworms as a fusion protein with HA + FLAG tags for DENV-1 and Strep-tag II + FLAG tags for DENV-4

DENV structural proteins are composed of a C, and two membrane proteins, prM and E translated at the beginning of the polyprotein in the order C-prM-E. BmNPV/1CprME (Fig. 1a), BmNPV/1prME (Fig. 1b), BmNPV/4CprME (Fig. 1c), and BmNPV/4prME (Fig. 1d) bacmids were injected into silkworm larvae, and the silkworm hemolymph was collected at 5 dpi. The expression of 1CprME, 1prME, 4CprME, and 4prME in hemolymph samples was confirmed, with molecular weights of 55 kDa at the E protein (Additional file 1: Fig. S1), which corresponded to the estimated weight.

### Purification of 1CprME, 1prME, 4CprME and 4prME polypeptides

**Fig. 2:**
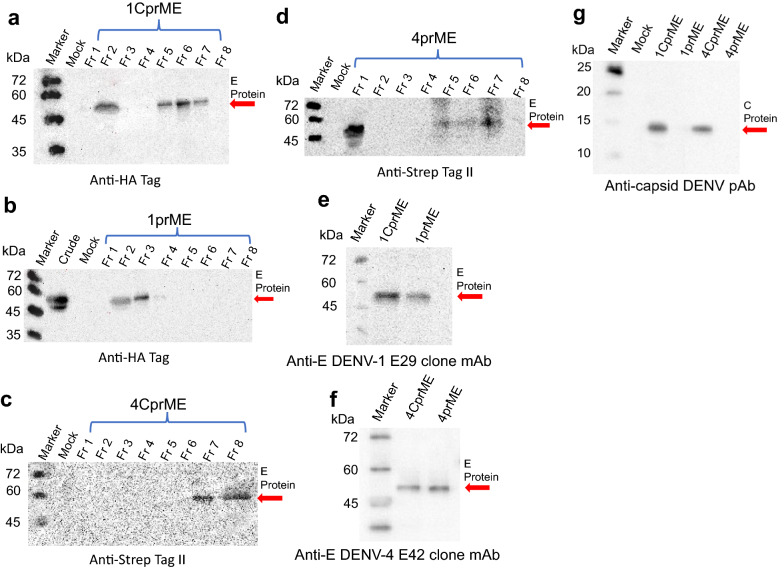
Western blot of purified (**a**) 1CprME, (**b**) 1prME, (**c**) 4CprME and (**d**) 4prME protein fractions (Fr). Each protein was purified from silkworm hemolymph using anti-FLAG tag protein purification gel column chromatography. E protein was verified using specific serotype monoclonal antibodies using (**e**) an anti-E E29 clone for DENV-1 constructs and (**f**) an anti-E E42 clone for DENV-4 antibodies. The C protein also confirmed using anti-capsid pAb (**g**)

**Fig. 3:**
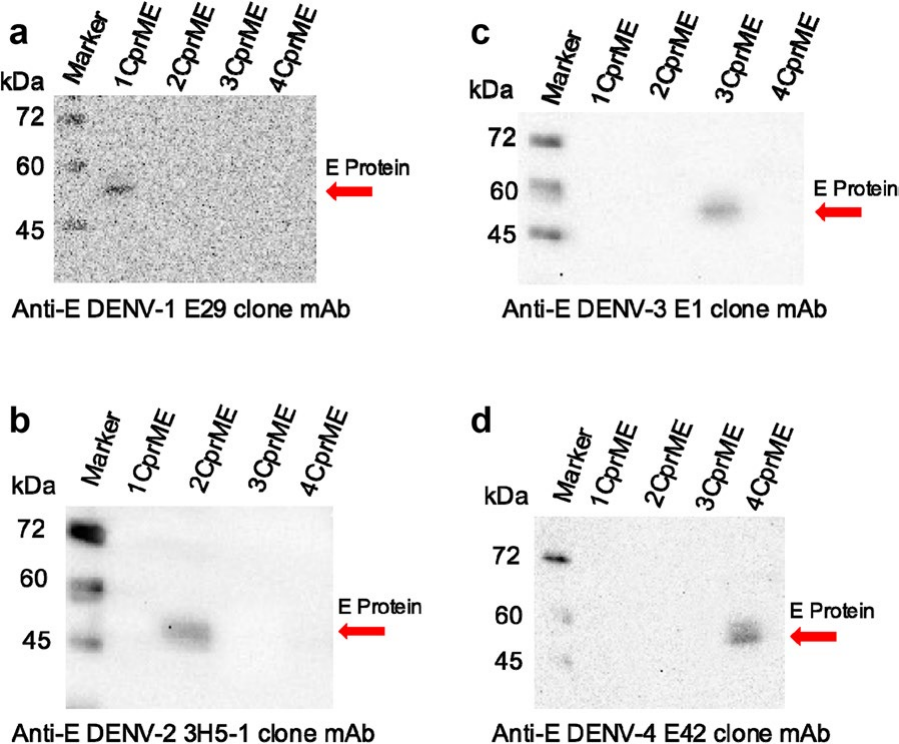
Western blot of purified (**a**) 1CprME, (**b**) 1prME, (**c**) 4CprME, and (**d**) 4prME polypeptides for the cross-reaction test was performed on the DENV-1 and DENV-4 constructs with specific serotype monoclonal antibodies for each serotype

The 1CprME, 1prME, 4CprME, and 4prME polypeptides were purified by affinity chromatography and confirmed by western blot using mouse anti-HA tag antibody for DENV-1 constructs (Fig. 2a, b) and anti-strap tag II antibody for DENV-4 constructs, which showed bands of size 55 kDa at elution fractions (Fr), respectively. To determine whether the purified 1CprME, 1prME, 4CprME, and 4prME polypeptides contained E proteins, western blotting was performed using serotype-specific monoclonal anti-envelope antibodies, an anti-E DENV-1 E29 clone for DENV-1 constructs, and an anti-E DENV-4 E42 clone for DENV-4 constructs. All of the constructs' bands were confirmed to be approximately 55 kDa (Fig. 2e, f). These results demonstrate that the E proteins were present in the purified 1CprME, 1prME, 4CprME, and 4prME polypeptides. The C protein was confirmed using anti-capsid pAb, which showed a band of size 14 kDa only in CprME constructs (Fig. 2g). Anti-E-DENV-1 E29 clone, anti-E-DENV-2 3H5-1 clone, anti-E-DENV-3 E1 clone, and anti-E-DENV-4 E42 clone antibodies were used to investigate the cross-reactivity of all purified proteins. Specific bands for 1CprME (Fig. 3a), 1prME (Fig. 3b), 4CprME (Fig. 3c), and 4prME (Fig. 3d) could not be detected using the specific serotype antibodies. These results indicate no cross-reactivity between specific serotype antibodies and all the constructs.

### Morphology of 1CprME, 1prME, 4CprME and 4prME polypeptides

**Fig. 4:**
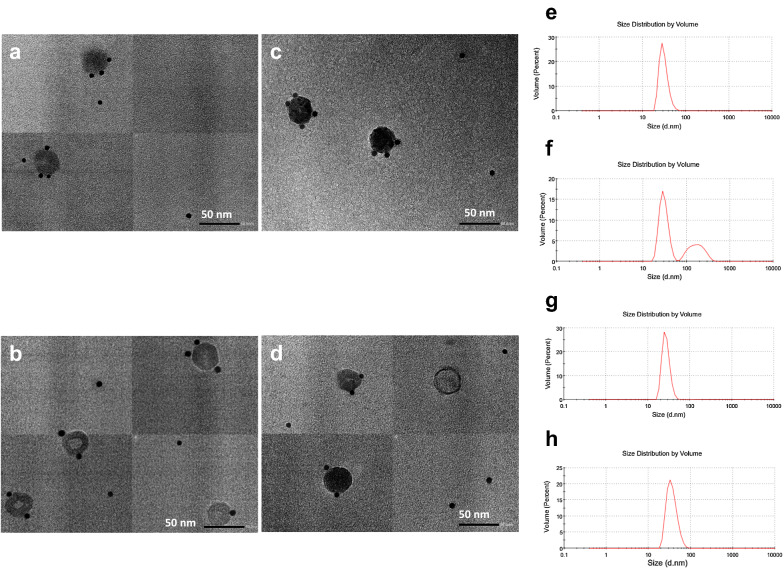
The purified (**a**) 1CprME, (**b**) 1prME, (**c**) 4CprME, and (**d**) 4prME polypeptides were immunogold labeled using an anti-E polyclonal antibody and analyzed by IEM. Black spots in A, B, C, and D indicate the immunogold label. Dynamic light scattering (DLS) was used to analyze the size distributions of (**e**) 1CprME, (**f**) 1prME, (**g**) 4CprME and (**h**) 4prME polypeptides

IEM was used to confirm the morphology of the polypeptides 1CprME, 1prME, 4CprME, and 4prME. Spherical structures with sizes ranging from 30 to 55 nm were observed (Fig. 4a–d), supported by data from dynamic light scattering (Fig. 4e–h). The IEM observation revealed that the particles were lipid bilayer-structured spherical, with some immunogold bound to their surface. The presence of anti-dengue E protein in gold nanoparticles bound to the surface of spherical structures indicates that the particles contain dengue E protein on the surface of the VLPs. According to these results, the 1CprME, 1prME, 4CprME, and 4prME polypeptides expressed in silkworms can generate VLPs of Dengue-1 and -4 (DENV-LPs/1CprME, DENV-LPs/1prME, DENV-LPs/4CprME, and DENV-LPs/4prME).

### Heparin-binding assay of the DENV-LPs/1CprME, /1prME, /4CprME, and /4prME

**Fig. 5:**
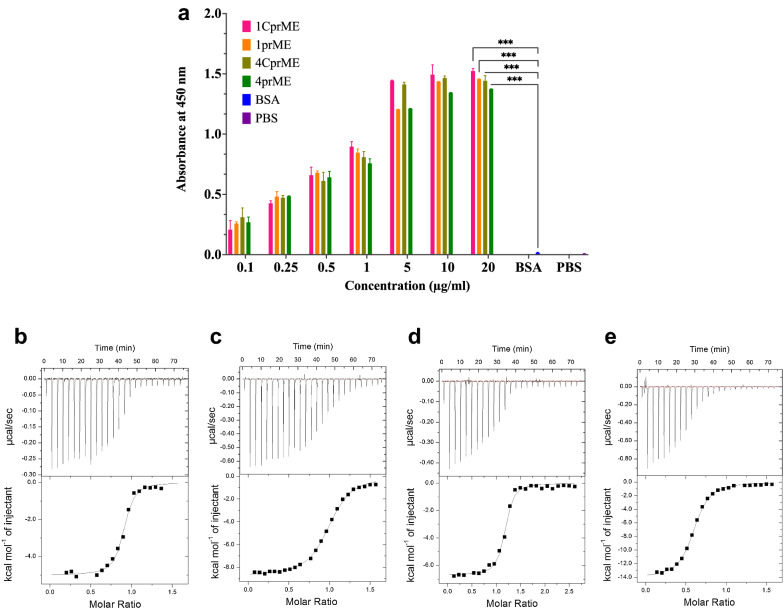
**a** Binding assay of DENV-LPs/1CprME, /1prME, /4CprME, and /4prME to heparin using ELISA. Heparin (1.8 ng) was coated onto each well of an ELISA plate, and each amount of purified protein was used for this ELISA, which was carried out according to the protocol described in the Materials and methods (Welch t-test, *p < 0.05, **p < 0.01, ***p < 0.001). **b**–**e** The binding activities between DENV-LPs/1CprME, /1prME, /4CprME, and /4prME toward heparin were examined by ITC

A heparin-binding assay was performed to confirm the expression of EDIII on the surface of DENV-LPs. The binding assay of the purified DENV-LPs/1CprME, /1prME, /4CprME, and /4prME was performed using heparin-immobilized microtiter plates. In ELISA, the absorbance increased proportionally to the presence of E (Fig. 5a). These results indicate that the EDIII domain is present on the surface of the DENV-LPs/1CprME, /1prME, /4CprME, and /4prME.

### Isothermal titration calorimetry assay for heparin-binding to DENV-LPs

Binding activities of DENV-LPs/1CprME, /1prME, /4CprME, and /4prME toward heparin were investigated by ITC (Fig. 5b–e). Heparin binds to DENV-LPs with *K*_D_ values of 197–952 nM and Δ*G* values from – 8.8 to – 9.3 kcal mol^–1^ (Table 2). *K*_D_ values, 235.2 nM of DENV-LPs/1CprME or 197.6 nM of /4CprME were 4-folds lower than 952.4 nM of DENV-LPs/1prME or 819.7 nM of /4prME, suggesting CprMEs are higher affinity to heparin than prMEs, which are the similar results of ELISA (Fig. 5a). Δ*G* value of DENV-LPs/1CprME or /4CprME was similar to DENV-LPs/1prME or /4prME, indicating that the heparin binding to the EDIII on DENV-LPs/CprMEs and DENV-LPs/prMEs has a similar spontaneously binding.

**Table 2:** The binding affinity of DENV-LPs towards heparin

Protein	*K*_D_ (nM)	*K*_a_ (M^–1^)	N	Δ*G* (kcal mol^–1^)	Ligand	Host	Method	Refs.
DENV-LP/1CprME	235.2	4.44 × 10^6^	0.88	– 8.92	Heparin	*B. mori*	ITC	This study
DENV-LP/1prME	952.4	1.05 × 10^6^	0.98	– 9.28	Heparin	*B. mori*	ITC	This study
DENV-LP/4CprME	197.6	5.06 × 10^6^	1.14	– 8.99	Heparin	*B. mori*	ITC	This study
DENV-LP/4prME	819.7	1.22 × 10^6^	0.58	– 8.85	Heparin	*B. mori*	ITC	This study
DENV-2 E protein	5.0	–	–	–	*O*-sulfated heparin	*E. coli*	SPR	Marks et al. (2001)
DENV-2 E protein	5.0	–	–	–	*N*- and *O*-sulfated heparin	*E. coli*	SPR	Marks et al. (2001
DENV-2 E protein	15.0	–	–	–	Heparin	COS-7	ITC	Chen et al. (1997)
ZIKV-E	433.0	–	–	–	Heparin	*E. coli*	SPR	Kim et al. (2017)

### Human DENV-infected sera interaction with DENV-LPs

**Fig. 6:**
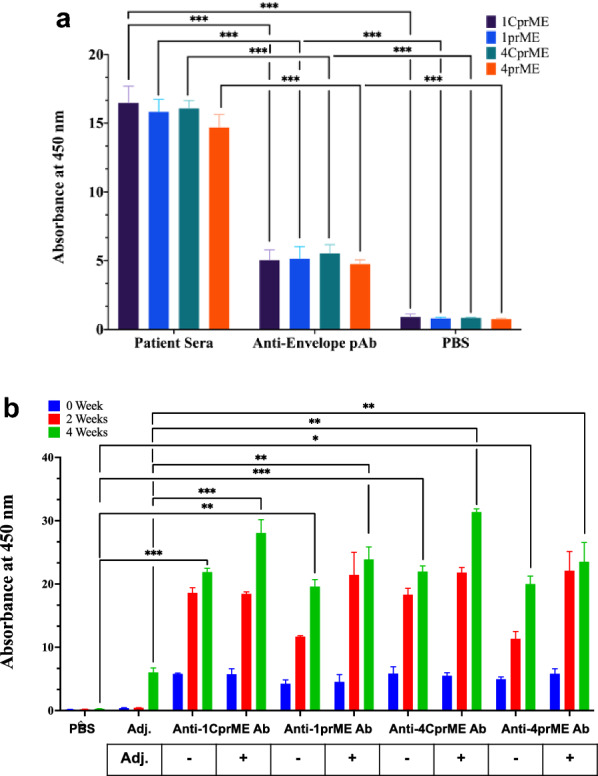
**a** The interactions between DENV-LPs/1CprME, /1prME, /4CprME and /4prME with mixed sera from dengue patients [rapid diagnostic test NS1(+)] were investigated (Welch t-test, *p < 0.05, **p < 0.01, ***p < 0.001). **b** Binding reactions were analyzed using direct ELISA as described. Specific IgG generation by DENV-LPs/1CprME, /1prME, /4CprME and /4prME. BALB/c mice were intraperitoneally immunized with 50 μg of monovalent DENV-LPs/1CprME, /1prME, /4CprME and /4prME. At 0, 2 and 4 weeks, sera were collected and used to test for binding of the specific IgG (Welch t-test, *p < 0.05, **p < 0.01, ***p < 0.001)

Purified DENV-LPs/1CprME, /1prME, /4CprME, and /4prME were characterized for their antigenicity toward the patient sera by direct ELISA. As shown in ELISA analysis, DENV-LPs/1CprME, /1prME, /4CprME, and /4prME showed reactivity to patient sera compared to PBS and the rabbit anti-DENV E polyclonal antibody as a positive control (Fig. 6a). DENV-LPs/1CprME, /1prME, /4CprME, and /4prME to patient sera showed significantly high responses compared to anti-E antibody. The Welch t-test showed a significant difference (p < 0.001) between DENV-LPs-patient sera reactivity to DENV-LPs-positive control or PBS.

### DENV-LPs/1CprME, /1prME, /4CprME and /4prME elicited virus-specific IgG

BALB/c mice were immunized three times at 3-week intervals with 50 μg of DENV-LPs/1CprME, /1prME, /4CprME, and /4prME with an alhydrogel adjuvant injected intraperitoneally. ELISA was used to measure serum titers in mice 2 weeks after their last immunization. DENV-LPs/1CprME, /1prME, /4CprME, and /4prME induced antibody production. The anti-1CprME Ab, anti-1prME Ab, anti-4CprME Ab, and anti-4prME Ab were tested for specific binding using their antigens. The anti-1CprME and anti-4CprME antibodies had high affinities for their respective DENV-LPs (Fig. 6b). Anti-1prME Ab and anti-4prME Ab, on the other hand, recognized DENV-LPs/1prME and DENV-LPs/4prME, respectively. Adjuvants increase immune responses to all antibodies, as shown in this study. Therefore, DENV-LPs/CprME are more effective at eliciting particular antibodies than DENV-LPs/prME.

## Discussion

Previously, DENV-LP/2 has expressed in silkworm larval hemolymph, and DENV-LP/3 was expressed in silkworm larval fat bodies by removing capsid-anchor in the capsid region (Utomo et al. 2019, 2020). However, the expressed 1CprME, 1prME, 4CprME, and 4prME polypeptides were detected in hemolymph by adding anchor c to the capsid region.

The interaction between heparin and DENV-LPs has been analyzed using multiple assays, including ELISA with immobilized heparin and ITC. Heparin binds to DENV-LPs with *K*_D_ values of 197–952 nM and Δ*G* values from – 8.8 to – 9.3 kcal mol^–1^ using the ITC system. According to other references, Marks et al. (2001) used *O*-sulfated heparin or *N*- and *O*-sulfated heparin to bind to the DENV-2 envelope protein expressed in *E. coli* and analyzed using SPR, yielding a *K*_D_ of 5 nM. Chen et al. (1997) used heparin to study the binding to the DENV-2 envelope protein expressed on COS-7 mammalian cells. They determined the *K*_D_ to be 15 nM using ITC. Kim et al. (2017) used heparin to analyze the binding of the ZIKV envelope protein expressed in *E. coli* and determined the *K*_D_ to be 433 nM using SPR (Table 2).

Compared to a protein subunit, VLPs are complex molecules composed of several protein subunits. It is known that the affinity of the ligand–protein is based on the surface's interaction, leading to the conjugation within the pocket of the protein. Hence, according to the structure of the VLP, it is considered a relatively high molar mass of macromolecules which affects the equilibrium and kinetics of its protein function. Consequently, the low effective molar concentration of a VLP results in the low surface available for binding and the overall affinity of a VLP binding being underestimated. Regarding antiviral activity, sulfated heparin has a better binding ability than heparin. Therefore, sulfated heparin is used to inhibit viral infection of cells. However, unmodified heparin in the cell membrane binds and interacts with EDIII, the putative receptor-binding domain in the flavivirus E protein crystal structure (Frei et al. 2018; Han et al. 2018; Yang et al. 2016; Zautner et al. 2006). EDIII also contains epitopes that block viral adsorption and are targeted by many antibodies, including serotype-specific neutralizing monoclonal antibodies. The conformational flexibility of heparin might permit this molecule to more easily adopt a productive conformation for interaction with the envelope protein. Although it has only a moderate binding affinity, the envelope of DENV-LP is still recognized by heparin on the surface of the cell (Hyatt et al. 2020; Marks et al. 2001). While mammalian cells secrete proteins with correct conformations and full biological activity, insect cells offer advantages compared to those of mammalian cells compared to *E. coli* or yeast expression systems. The ability to introduce foreign DNA into these cells facilitates a better understanding of the transcriptional, translational, and posttranslational machinery in mammalian cells (Gray 2001; Ikonomou et al. 2003).

The antigens of 1CprME, 1prME, 4CprME, and 4prME also showed reactivity to patient sera high affinity in the direct ELISA. Mixed sera of dengue patients can react to many types of nonspecific DENV epitopes. The strongly correlated reactivities of patient sera with DENV-LPs indicated that the same epitope(s) were displayed on these DENV-LPs. However, there will be a marked difference in the level of antibody reactivity. In other words, the epitope(s) displayed on the DENV-LP surface is comparable with those of the native dengue virus (Danko et al. 2018; Wang et al. 2003).

DENV-LPs/1CprME, /1prME, /4CprME, and /4prME could generate IgG antibody levels since many VLPs include structural or molecular characteristics that give certain auto-immunostimulatory qualities. These features enable VLPs to induce immunological responses without adjuvants. Nevertheless, adjuvants may enhance vaccination immunogenicity and encourage activating a specific type of immune response when combined with VLP vaccines (Cimica and Galarza 2017; Donaldson et al. 2018; Müller et al. 2020). Our ITC data indicate that DENV-LP/1CprME and DENV-LPs/4CprME have a lower *K*_D_ than DENV-LP/1prME and DENV-LPs/4prME. This demonstrates that the CprME constructs have a higher affinity binding than the prME constructs. Those reactions can be harnessed to perform work inside the body. The IgG elicitation in mice confirmed DENV-LP/CprMEs elicit a stronger immune response than DENV-LPs/prMEs, which indicates CprME can easily bind to a heparin-like receptor on the surface of the cells compared to prME. This is supported by the results on heparin-binding assays, as the CprME has 4 times lower *K*_D_ value than prME. This also indicates that the EDIII might be displayed well on the envelope region of the DENV-LP/CprMEs, so it can provide better activity in provoking an immune response in mice.

Furthermore, including capsid protein in the VLP design might also provide an additional antigen and better immunogenicity. When mice were immunized with flavivirus VLPs, CprME VLPs exhibited superior antigenicity to prME VLPs. Due to the superiority of the CprME VLP, a capsid should be included in the vaccine to improve immunity. This repetitive protein structure can boost innate immunity and prompt B cells to directly generate neutralizing antibodies (Garg et al. 2019; Nooraei et al. 2021). Each DENV serotype carries the conserved antibody epitope incorporated in the N- and C-terminal regions of the C protein and is efficiently recognized by dengue patients exposed to primary and secondary infections from other serotypes. The C-protein central region has an epitope of the peptide, primarily targeted by serotype-specific antibodies (Alves et al. 2016; Nadugala et al. 2017; Rana et al. 2018).

## Supplementary Information

**Additional file 1: Fig. S1.** Expression of 1CprME, 1prME, 4CprME and 4prME polypeptides in silkworm larvae.

## Data Availability

All the data and materials have been provided in the main manuscript.

## Abbreviations

Ab: Antibody
C: Capsid protein
DENV: Dengue virus
DENV-LP: Dengue virus-like particle
*K*_a_: Association constant
*K*_D_: Dissociation constant
DLS: Dynamic light scattering
E: Envelope protein
ELISA: Enzyme-linked immunosorbent assay
EDIII: Envelope protein domain III
Δ*G*: Gibbs free energy
HRP: Horseradish peroxidase
IEM: Immunoelectron microscopy
ITC: Isothermal Titration Calorimetry
M: Membrane protein
PBS: Phosphate-buffered saline
PBST: Phosphate-buffered saline containing 0.1% Tween 20
PCR: Polymerase chain reaction
prM: Premembrane protein
TBS: Tris-buffered saline
TBST: Tris-buffered saline containing 0.1% Triton X-100
TEM: Transmission electron microscopy
TMB: 3.3′,5.5′-Tetramethylbenzidine
VLP: Viral-like particle
WHO: World health organization
