# Supplementary material for: Humoral immune response induced with dengue virus-like particles serotypes 1 and 4 produced in silkworm
Source: AMB Express. 2022 Jan 31;12:8. doi: 10.1186/s13568-022-01353-6 (PMC8802989; doi:10.1186/s13568-022-01353-6)
Supplement: Supplementary file 1 — Additional file 1: Fig. S1. Expression of 1CprME, 1prME, 4CprME and 4prME polypeptides in silkworm larvae. [file 13568_2022_1353_MOESM1_ESM.docx]

**Additional file 1**

**Humoral immune response induced with dengue virus-like particles serotypes 1 and 4 produced in silkworm**

**Doddy Irawan Setyo Utomo^1^ · Sabar Pambudi^2^ · Enoch Y Park^1,3,^^[[1]](#footnote-1)^**

E-mails:

[doddy.irawan.setyo.utomo.16@shizuoka.ac.jp](mailto:doddy.irawan.setyo.utomo.16@shizuoka.ac.jp) (DISU)

[sabar.pambudi@bppt.go.id](mailto:sabar.pambudi@bppt.go.id) (SP)

[park.enoch@shizuoka.ac.jp](mailto:park.enoch@shizuoka.ac.jp) (EYP)


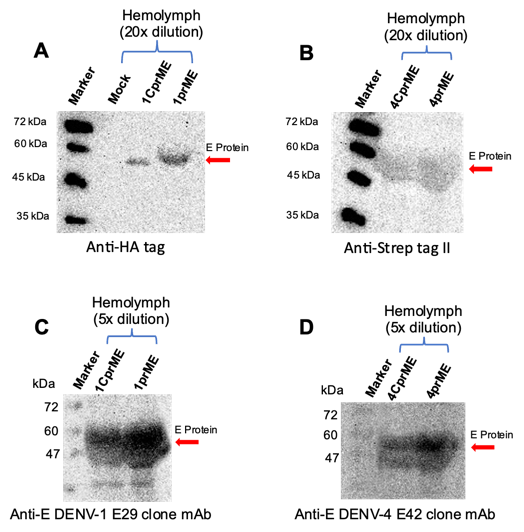


**Supplementary Figure S1**. **Expression of 1CprME, 1prME, 4CprME and 4prME polypeptides in silkworm larvae.** In the case of silkworm larvae, hemolymph were collected after recombinant BmNPV infection. The homogenate of each sample was prepared according to the protocol described in Materials and methods. (**A**) Expressed 1CprME, and 1prME polypeptides were detected by western blot using mouse anti-HA tag, (**B**) while 4CprME and 4prME polypeptides were detected by western blot using mouse anti-strep tag II as a primary antibody. To determine whether the purified 1CprME, 1prME, 4CprME, and 4prME polypeptides contained E proteins, western blotting was performed using specific serotype monoclonal anti-envelope antibodies, (**C**) anti-E DENV-1 E29 clone for DENV-1 constructs, and (**D**) anti-E DENV-4 E42 clone for DENV-4 constructs.

1. 🖂 Enoch Y. Park

   [park.enoch@shizuoka.ac.jp](mailto:park.enoch@shizuoka.ac.jp) (E.Y. Park)

   ^1^ Laboratory of Biotechnology, Department of Bioscience, Graduate School of Science and Technology, Shizuoka University, 836 Ohya, Suruga-ku, Shizuoka 422-8529, Japan

   ^2^ Center of Pharmaceutical and Medical Technology, National Research and Innovation Agency (BRIN), Jl. Kawasan Puspiptek, Gedung I LAPTIAB, Kota Tangerang Selatan, Banten 15314, Indonesia

   ^3^ Laboratory of Biotechnology, Research Institute of Green Science and Technology, Shizuoka University, 836 Ohya, Suruga-ku, Shizuoka 422-8529, Japan [↑](#footnote-ref-1)
